# Living on the Edge: Settlement Patterns by the Symbiotic Barnacle *Xenobalanus globicipitis* on Small Cetaceans

**DOI:** 10.1371/journal.pone.0127367

**Published:** 2015-06-17

**Authors:** Juan M. Carrillo, Robin M. Overstreet, Juan A. Raga, Francisco J. Aznar

**Affiliations:** 1 Department of Coastal Sciences, University of Southern Mississippi, Ocean Springs, Mississippi, United States of America; 2 Cavanilles Institute of Biodiversity and Evolutionary Biology, Science Park, University of Valencia, Paterna, Valencia, Spain; Duke University Marine Laboratory, UNITED STATES

## Abstract

The highly specialized coronulid barnacle *Xenobalanus globicipitis* attaches exclusively on cetaceans worldwide, but little is known about the factors that drive the microhabitat patterns on its hosts. We investigate this issue based on data on occurrence, abundance, distribution, orientation, and size of *X*. *globicipitis* collected from 242 striped dolphins (*Stenella coeruleoalba*) that were stranded along the Mediterranean coast of Spain. Barnacles exclusively infested the fins, particularly along the trailing edge. Occurrence, abundance, and density of *X*. *globicipitis* were significantly higher, and barnacles were significantly larger, on the caudal fin than on the flippers and dorsal fin. Barnacles were found more frequently and in greater numbers on the dorsal rather than ventral side of the caudal fin and on the central third of dorsal and ventral fluke surfaces. Nearly all examined individuals attached with their cirral fan oriented opposite to the fluke edge. We suggest that *X*. *globicipitis* may chemically recognize dolphins as a substratum, but fins, particularly the flukes, are passively selected because of creation of vortices that increase contact of cyprids with skin and early survival of these larvae at the corresponding sites. Cyprids could actively select the trailing edge and orient with the cirri facing the main direction of flow. Attachment on the dorsal side of the flukes is likely associated with asymmetrical oscillation of the caudal fin, and the main presence on the central segment of the flukes could be related to suitable water flow conditions generated by fluke performance for both settlement and nutrient filtration.

## Introduction

Several groups of symbiotic barnacles have been reported to settle on living host organisms, including sponges, cnidarians, molluscs, crustaceans, fishes, turtles, and cetaceans [1]. However, the type of interaction between a barnacle and its host varies. Many barnacles use organisms similarly to the way they use inanimate structures, and thus can be considered facultative epibionts [2] that lack specific adaptations to dwell on or in specific living organisms. For instance, the pedunculate barnacle *Conchoderma virgatum* is a fouling species typically found on flotsam, but it is also able to colonize the carapace of marine turtles (e.g., [3]) or even fishes, copepods on fishes [4, 5], and the teeth of dolphins [6] because these hosts offer a hard substratum on which to attach. Other barnacles, however, are obligate epibionts with specific adaptations to successfully detect, attach, feed and reproduce on living hosts [1, 2]. For instance, evidence shows that whale barnacles of the family Coronulidae are able to detect the presence of sessile barnacles on whales [5] or whale skin directly [7] and have a highly modified attachment device to overcome constant shedding of the host epidermis [8, 9].

Microhabitat selection of obligate epibiont barnacles on their hosts is peculiar in that each individual host represents a replicated patch for attachment, and therefore, it offers a predictable set of conditions. However, habitat selection is a behavioral process that requires identification of the spatial scale(s) at which decisions of the animals are made [10]. In this context, patterns of water that flow over the host are expected to be a major determinant of the overall barnacle distribution because the filtration system of barnacles largely depends on external currents to trap food [1]. Moreover, an optimal microhabitat should provide conditions for efficient filtration, but it also minimizes the negative consequences of drag on the physical integrity of barnacles [11]. In large, fast-swimming hosts, e.g., turtles and cetaceans, the hydrodynamic pattern is characterized by relatively intense currents with a predominant swimming direction; thus, the large-scale distribution of barnacles on these hosts should be determined by flow-water dynamics. Indeed, studies on cheloniibid barnacles from marine turtles suggest that barnacles primarily select areas of moderate flow, which allow optimal foraging and growth (e.g., [12, 13]). In the only such study on whale barnacles to our knowledge, Kasuya and Rice [14] speculated that the distribution and orientation of the coronulid *Cryptolepas rhachianecti* on two individuals of the gray whale, *Eschrichtius robustus*, follow the direction of water currents generated by the whales.

*Xenobalanus globicipitis* is an obligate cetacean barnacle that infests 34 species worldwide, particularly dolphins from tropical and temperate waters [15]. Like other coronulids, *X*. *globicipitis* appears to be able to react to chemical cues to identify suitable hosts, attaching to them using a reduced basal shell that penetrates into the host’s skin and produces wedge forces [8, 9]. Interestingly, *X*. *globicipitis* colonizes hosts that produce the most intense currents experienced by any obligate barnacle, attaching on swimming appendages, i.e., flukes, dorsal fin, and flippers, most often along the trailing edge ([15–17]; see [18] for exceptional records on other sites). Seilacher [8] speculated that fins are suitable habitats to take advantage of water current flows, but, other than his study, we know of no other quantitative account of microhabitat selection patterns of *X*. *globicipitis*. From wild bottlenose dolphins (*Tursiops truncatus*) Bearzi and Patonai [16] reported a higher occurrence and abundance of *X*. *globicipitis* on the upper segment of the dorsal fin when compared with medium and basal segments, but they provided no explanation for this distribution pattern.

In this paper, we investigate patterns of microhabitat selection of *X*. *globicipitis* on the striped dolphin, *Stenella coeruleoalba*, based on detailed data of occurrence, abundance, distribution, orientation, and size of barnacles at several spatial scales. Results are interpreted according to the factors that may affect recruitment, survival, and growth of individuals, paying special attention to the swimming performance of its hosts.

## Material and Methods

### Samples

Data were collected from 242 striped dolphins with an intact skin (carcass conservation codes 1–2 sensu [19]) found stranded along 556 km of coastline, from 40°101 31.5’N, 0° 31.0’ E to 37° 50.7’N, 1° 37.5’ W of Mediterranean coast of Spain between January 1979 and August 2009. Permission and funding to collect stranded dolphins was given by the Wildlife Service of the Valencian Regional Government, Spain, which is the official institution in charge of managing and protecting wildlife in the region. No ethics board was involved because animals were collected after their natural death. Some carcasses were examined for *X*. *globicipitis* on the beach, but most of them were brought to the laboratory for a more detailed analysis. The amount of data gathered varied over the years according to the human and economic resources available. Thus, sample sizes are given for each specific analysis.

**Fig 1:**
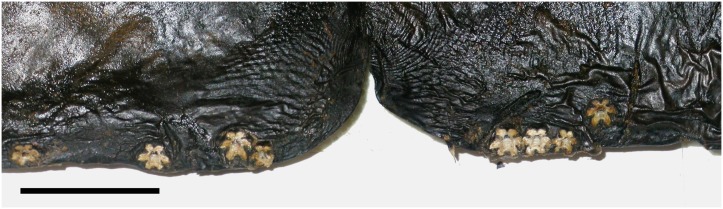
Basal shells of *Xenobalanus globicipitis*. Shells attached on the dorsal side of the flukes, close to the notch, of the striped dolphin, *Stenella coeruleoalba*. Scale bar: 2 cm.

Dolphins were measured to the nearest 0.5 cm, then carefully examined for epizoic crustaceans. The body stalk of *X*. *globicipitis* usually detaches when the host animal desiccates, and individuals are often detected by the presence of their basal shells that generally remain intact (Fig 1). Maximum shell diameter (MSD) has been shown to have a strong relationship with body-size and reproductive state of barnacles [6, 20] and therefore was used as an indicator of the size/age of each individual. For each individual, MSD was measured with a digital caliper to the nearest 0.1 mm based on *in situ* individuals or photographs of them.

The dolphin sample contained a substantial amount of individuals that were killed by a morbillivirus in 1990 and 2007 [21]. There is evidence that the disease increased the probability of settlement of *X*. *globicipitis* because of slow swimming behavior and immunosuppression [6, 20]. Thus, in all analyses, we compared habitat selection patterns in epizootic vs. non-epizootic samples to ensure that the illness did not alter habitat selection patterns.

### Patterns of occurrence, density and size of *X*. *globicipitis* between fins

**Fig 2:**
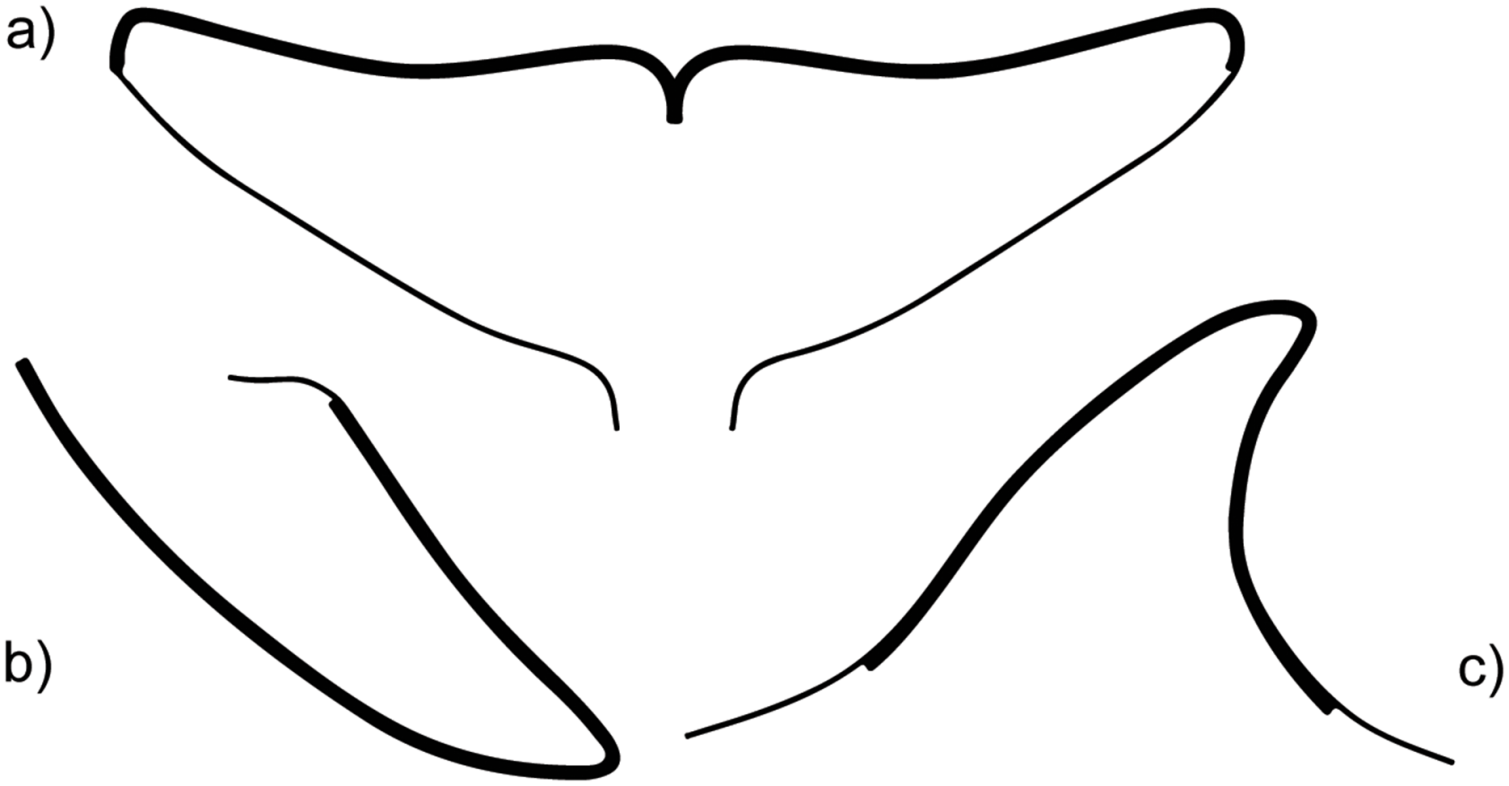
Area colonized by *Xenobalanus globicipitis*. Diagram of dolphin flukes (a), flipper (b), and dorsal fin (c), highlighting the area where individuals of *X*. *globicipitis* were found on the striped dolphin, *Stenella coeruleoalba*.

*Xenobalanus globicipitis* appeared almost exclusively on the trailing edge of dorsal fin, flippers, and flukes covering a strip of approximately 2 cm wide in all fins (Fig 2). We tested the null hypothesis of random colonization among fins according to their size. To determine the probability of colonization of each fin, we initially assumed that the width of the area colonized was similar among fins (approximately 2 cm). We then measured the perimeter of the dorsal fin, the flippers, and the flukes to the nearest 0.1 cm (Fig 2) with the software ImageTool 3.0 [22] based on digital photographs of all fins in lateral view from 45 dolphins. Limits of perimeter measurements were set based on the distribution of *X*. *globicipitis* in the overall sample of dolphins, i.e., their fundamental niche. The average perimeter of each fin (i.e., dorsal fin, left + right flippers, flukes) was transformed into a probability value as p_i_ = A_i_/AT, where A_i_ is the perimeter of fin *i* and AT is the summed perimeter of all fins. A chi-square test was used to test the goodness of fit between the observed occurrences and the expected occurrences according to the null hypothesis. Only dolphins positive for *X*. *globicipitis* (n = 94) were included in the analysis. To generate 95% confidence intervals (CI) for occurrence on each fin under the null hypothesis, we generated 20,000 random matrices using EcoSim 7 [23] as follows. Observed row incidence totals (i.e., the number of colonized fins in each dolphin) were fixed, and columns (fins) were filled randomly according to the probabilities calculated above [24]. The 95% CIs were obtained by removing values below and above the 2.5% and 97.5% percentiles, respectively.

Abundance of *X*. *globicipitis* per fin was obtained from 78 dolphins and tested for significant differences of abundance between fins using a Friedman test with *post hoc* comparisons [25]. Out of the 45 dolphins for which fin perimeter was calculated, 31 harbored *X*. *globicipitis*. We calculated linear density as the number of individuals per fin divided by fin perimeter (Fig 2); values were tested for significant differences of density between fins using the Friedman test.

Permutational multivariate analysis of variance (PERMANOVA) based on a similarity matrix [26] was used to test whether there were differences in abundance of *X*. *globicipitis* per fin between ‘epizootic’ (n = 36) and ‘non-epizootic’ (n = 42) dolphins. To build the model, raw abundance data per fin was square-root transformed, and a Bray—Curtis similarity matrix between dolphins was obtained. Pseudo-F statistics under a true null hypothesis were obtained by using a permutation procedure, i.e., group labels were randomly shuffled onto different sample units, and this procedure was repeated 20,000 times (see [26] for details).

To gain insight about the effect of the time of recruitment (which is obviously uncontrolled) and habitat suitability on the size of *X*. *globicipitis*, we measured the maximum diameter of basal shell of 994 individuals from 59 dolphins and calculated median values per fin in each individual dolphin. Then, a general linear mixed model (GLMM) using restricted maximum likelihood was built. GLMM is a flexible procedure that allows us to estimate unbiased parameters even with unbalanced, correlated data [27]. Median value of shell-size was used as the dependent variable, ‘fin’ and ‘dolphin type’ (‘epizootic’ vs. ‘non-epizootic’) were included as fixed factors, and ‘dolphin individual’ as a random factor. To control for potential density-dependent effects, we included ‘barnacle abundance per fin’ as a fixed covariate. We initially included interaction terms, but none were statistically significant, so we removed all to increase the sensitivity of the analysis and to correctly interpret main effects [28]. All analyses were performed with the statistical package SPSS v. 19.

### Patterns of distribution, density, size, and orientation of *X*. *globicipitis* on the flukes

**Fig 3:**
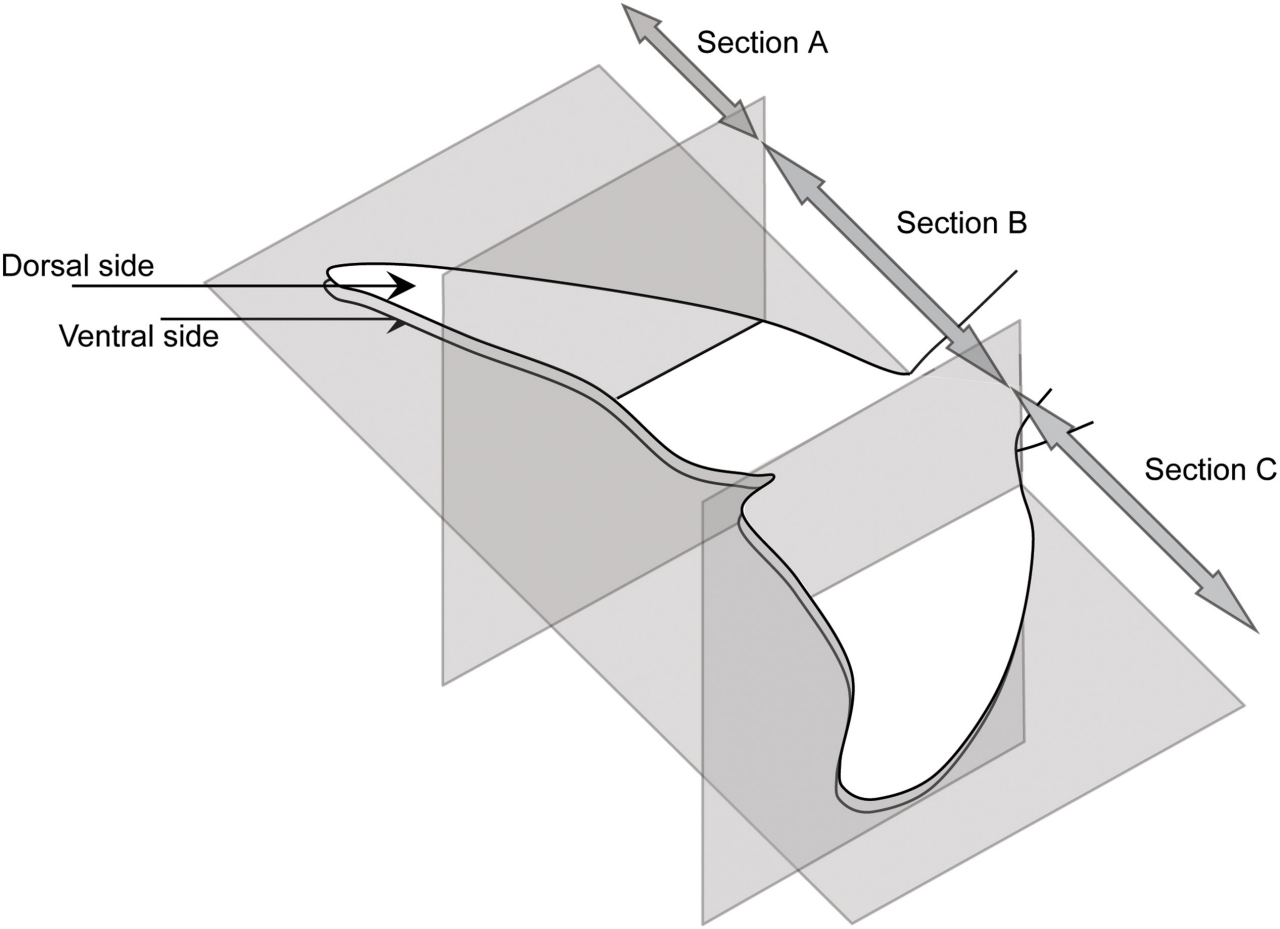
Microhabitats. Microhabitats defined for the study of the habitat selection for *Xenobalanus globicipitis* on the flukes of the striped dolphin, *Stenella coeruleoalba* (see the text for details).

A more refined analysis of habitat selection was conducted on the caudal fin because it was by far the most frequently occupied microhabitat. The flukes of 45 dolphins positive for *X*. *globicipitis* were divided into three transversal segments of equal length by dividing the standard length of the flukes, i.e., the maximum distance from tip to tip (Fig 3). Given that the rear perimeter of the flukes is, for the most part, straight, we assumed that these three segments represented microhabitats of roughly similar size. Individuals of *X*. *globicipitis* were then counted on the dorsal and ventral sides of each defined segment. We used Wilcoxon test to compare the overall number of *X*. *globicipitis* between sides and Friedman test with *post hoc* comparison to test for significant differences between the three segments on both the dorsal and ventral sides of the flukes.

A PERMANOVA test based on a similarity matrix [26] was used to determine whether the pattern of abundances of *X*. *globicipitis* on the six defined sites (i.e., 3 segments per side) differed between ‘epizootic’ (n = 19) and ‘non-epizootic’ (n = 26) dolphins.

Maximum shell diameter of *X*. *globicipitis* was obtained for individuals of each of the six sites defined per side and segment. A GLMM was built using, as the dependent variable, the median value of shell per site for each individual dolphin. ‘Segment’, ‘side’, and ‘dolphin type’ (‘epizootic’ vs. ‘non-epizootic’) were used as fixed factors, ‘dolphin individual’ as a random factor, and ‘barnacle abundance per side and segment’ as a fixed covariate. Two-order interactions were initially included in the model and eventually removed because they were not significant [28].

**Fig 4:**
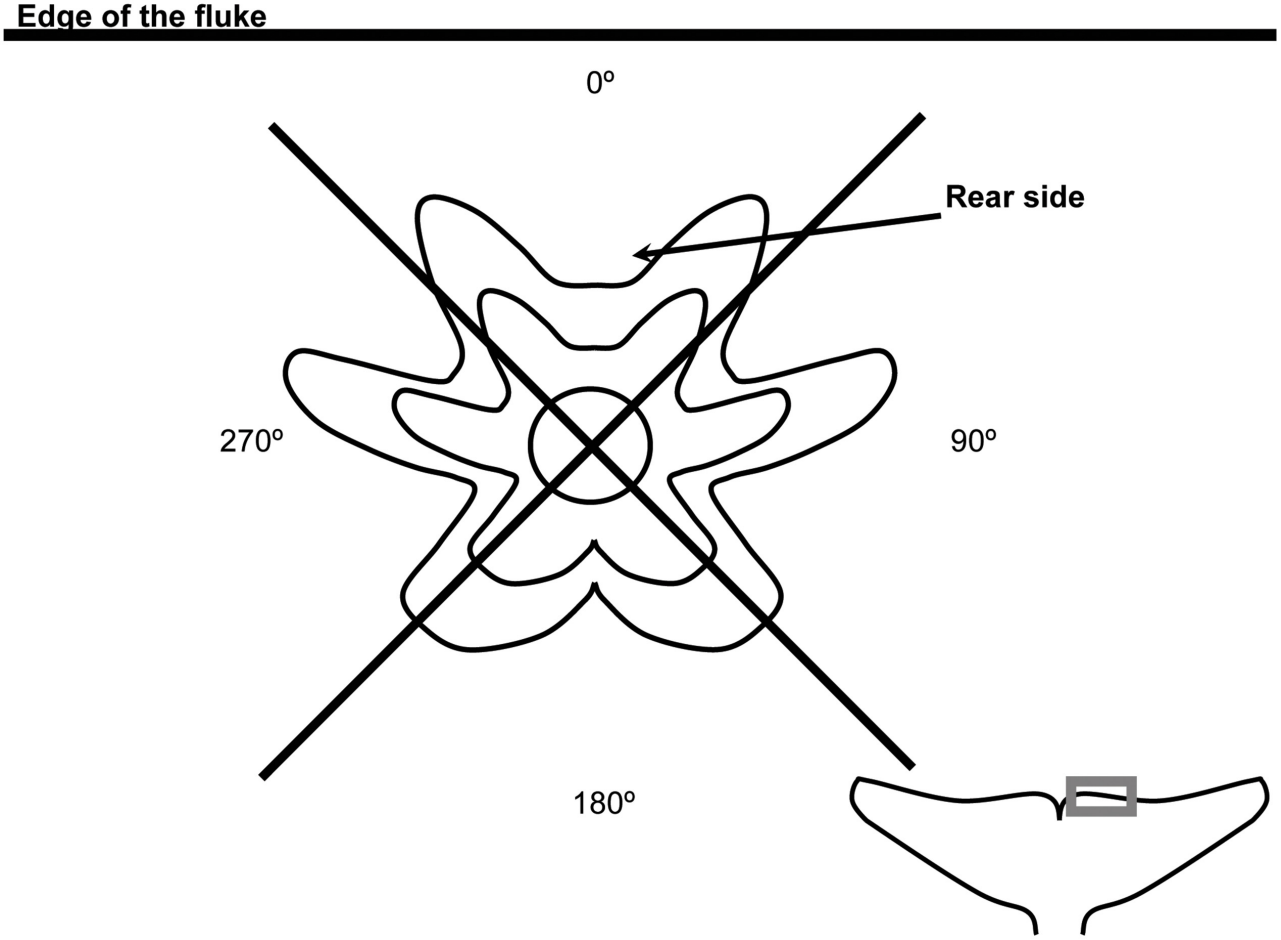
Orientation criterion for the basal plate. Diagram of a basal plate of *Xenobalanus globicipitis* and criterion used to orient it with respect to the edge of the fluke of the striped dolphin, *Stenella coeruleoalba* (see the text for details).

Based on observations of 34 intact individuals of *X*. *globicipitis*, we determined that the cirral fan was always oriented opposite to the rear side of the basal plate, i.e. towards the convex part of the shell (Fig 4). For 63 barnacles from 21 dolphins, we recorded the orientation of the rear side of the shells with respect to fluke edge using four quadrants (Fig 4).

Sterne’s exact 95% CI (see [29]) was calculated for overall percent occurrence of *X*. *globicipitis* in the sample of dolphins. Given that the population of *X*. *globicipitis* was aggregated, a bootstrap procedure with 20000 replications was used to set 95% C.I.s of mean linear density and mean abundance of *X*. *globicipitis* in specific microhabitats (see [30] for details). All these analyses were performed with the software Quantitative Parasitology 3.0 [31].

## Results and Discussion

### Results

*Xenobalanus globicipitis* was detected on 104 of 242 dolphins (43.0%; 95% CI: 36.8–49.4). Barnacles appeared on the trailing edge, more rarely on the leading edge of dorsal fin and flippers, and exclusively on the trailing edge of flukes, covering a strip of approximately 2 cm wide on all fins (Fig 2). The number of barnacles was counted on 93 dolphins, the average number per dolphin being 18.2 (CI 95%: 13.8–24.7), with a median value of 9 (95% CI: 6–11) and a range of 1–132 individuals. Variance-to-mean ratio (including uninfected hosts) was 49.3, indicating a highly aggregated distribution.

#### Patterns of occurrence, density, and, size of *X*. *globicipitis* between fins

**Fig 5:**
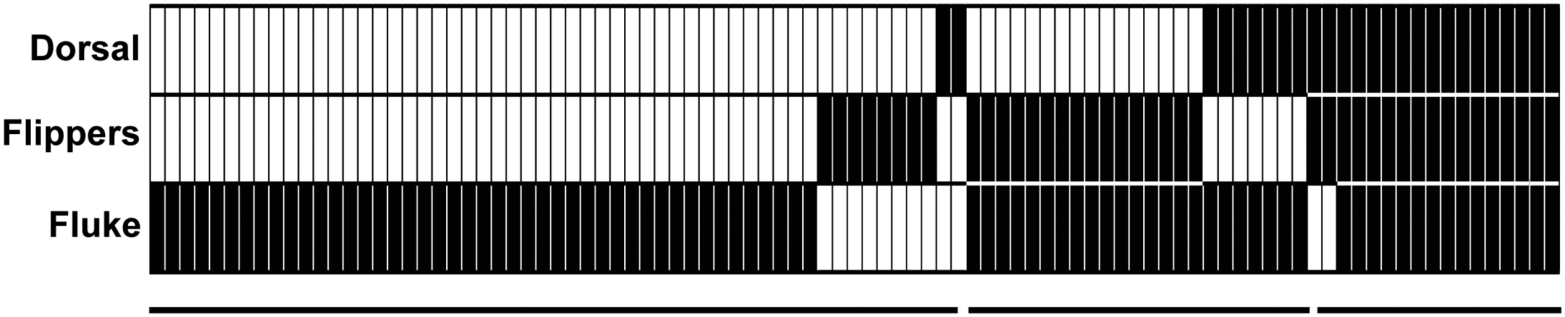
Pattern of occurrence of *Xenobalanus globicipitis* on swimming appendages using the criterion of maximum nesting. Each column shows an individual striped dolphin, *Stenella coeruleoalba*, and the presence of the barnacle on each appendage is represented by a black rectangle. The bars below indicate the number of appendages colonized.

**Fig 6:**
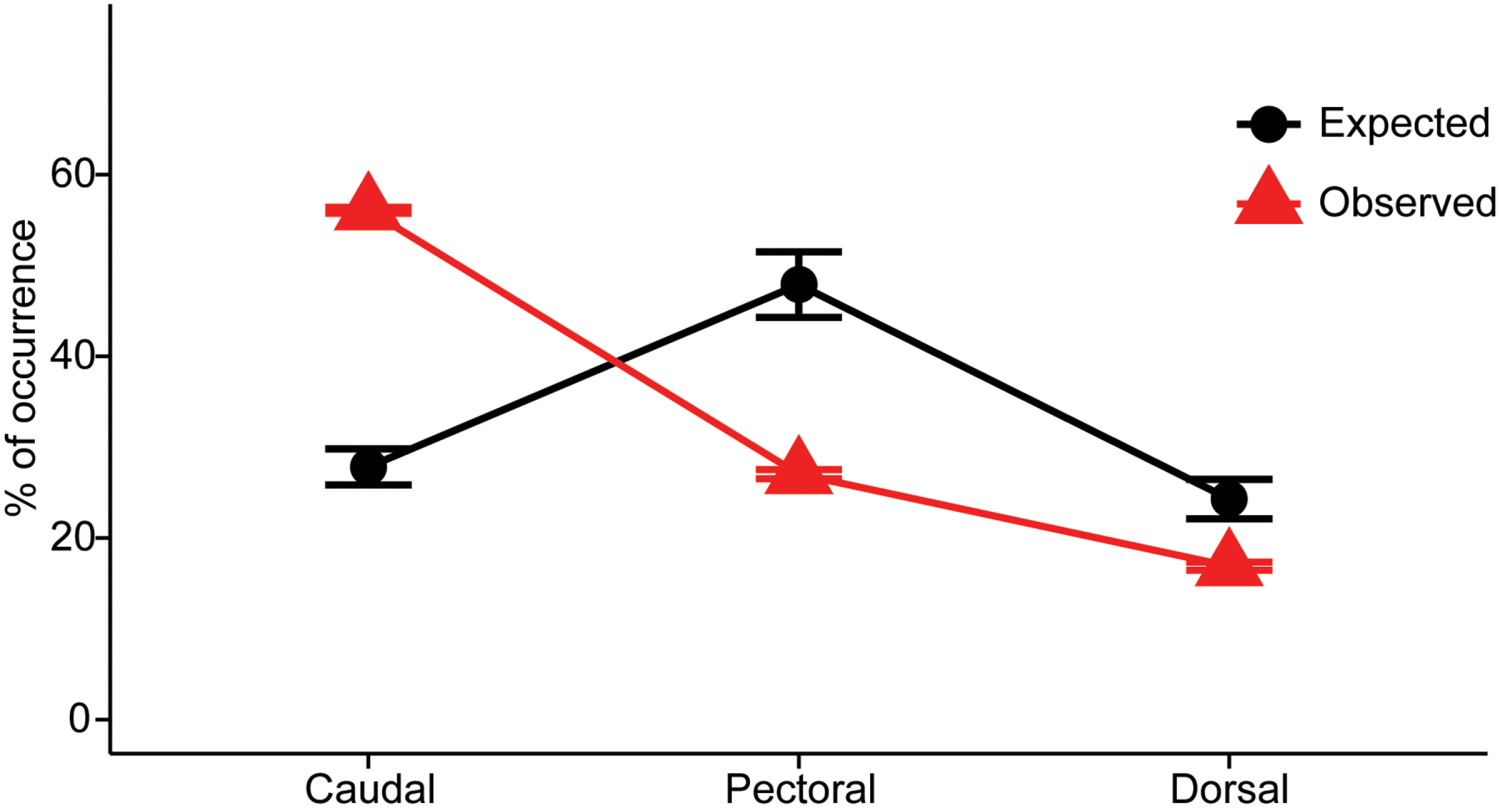
Percent occurrence of *Xenobalanus globicipitis* on flukes, flippers, and dorsal fin of 96 striped dolphins (*Stenella coeruleoalba*). Triangles indicate actual figures, and circles indicate expected figures in a total of 96 striped dolphins, *Stenella coeruleoalba*, assuming that the probability of colonization depends on space provided on each fin (see text for details). Bars represent the 95% confidence interval.

Data on occurrence of *X*. *globicipitis* (n = 95 dolphins) indicate that the flukes were by far the most frequent site of occurrence, followed by flippers and dorsal fin in a clear nested pattern (Fig 5). The average space (± SD) for settlement (cm) was as follows: flukes, 48.2 (9.6); each flipper, 40.6 (6.2); and dorsal fin, 40.1 (5.1). Thus, the probability of occurrence according to available space for settlement was 0.284 (flukes), 0.479 (flippers) and 0.237 (dorsal fin). However, the frequency of occurrence was significantly higher on the flukes and lower on flippers and dorsal fin than values expected, according to available space (chi-square test: χ^2^ = 34.32, 2 d.f., p< 0.0001; Fig 6).

Data on the number of barnacles per fin were available from 84 dolphins positive for *X*. *globicipitis*. The average number (95% CI) was as follows: flukes, 11.3 (8.1–16.8); flippers, 6.4 (3.5–11.6); and dorsal fin, 0.8 (0.4–1.6). Differences of abundance were significant (Friedman test, χ^2^ = 63.03, 2 d.f., p<0.0001) as were all *post hoc* comparisons between fins (p<0.01). Linear density (no. barnacles/cm) obtained from 65 dolphins also significantly differed between fins: average was 0.27 on flukes; 0.19 on flippers, and 0.01 on dorsal fin (χ^2^ = 57.43, 2 d.f., p<0.0001; all *post hoc* comparisons <0.01). A logistic regression indicated that the occurrence of *X*. *globicipitis* on the flippers and on the dorsal fin was not related to density on the flukes (Wald statistic = 0.091, 1 d.f., one-tailed p = 0.381).

The PERMANOVA test indicated that the pattern of abundances among fins did not significantly differ between ‘epizootic’ and ‘non-epizootic’ dolphins (F_(1,76)_ = 0.935, p = 0.408).

The average (SD) median shell diameter (mm) of *X*. *globicipitis* was 2.66 (1.33) on the flukes (n = 50 dolphins); 2.21 (0.91) on flippers (n = 23); and 2.56 (0.94) on the dorsal fin (n = 9). Results from the mixed model indicated that ‘fin’ (F_(2, 48.17)_ = 3.340, p = 0.044), and ‘log_10_-abundance’ (F_(1, 74.42)_ = 5.185, p = 0.026), but not ‘dolphin type’ (i.e., ‘epizootic’ vs. ‘non-epizootic’) (F_(1, 48.02)_ = 3.340, p = 0.044) were significant predictors of median shell diameter. Parameter estimation is shown in Table 1. Populations of *X*. *globicipitis* from flippers and the dorsal fin had smaller shells when compared with those on the flukes; the difference was significant in the flukes-flippers comparison. Also, shell size decreased at higher population sizes (Table 1).

**Table 1:** Parameters of predictors in a mixed model that accounts for the median shell diameter of *Xenobalanus globicipitis* on the flukes, flippers and dorsal fin of the striped dolphin, *Stenella coeruleoalba*, from the western Mediterranean Sea.

Parameter	Estimation	S.E.	d.f.	t	P
Constant	3.167	0.281			
Fin					
*Flippers*	-0.307	0.318	51.511	-0.966	0.339
*Dorsal*	-0.564	0.220	47.147	-2.567	0.013
*Flukes*	0	0	-	-	-
Log intensity	-0.603	0.265	76.026	-2.277	0.026

Parameters for ‘flippers’ and ‘dorsal fin’ were obtained by setting that of ‘flukes’ to zero.

#### Patterns of distribution, density, size, and orientation of *X*. *globicipitis* on the flukes

**Fig 7:**
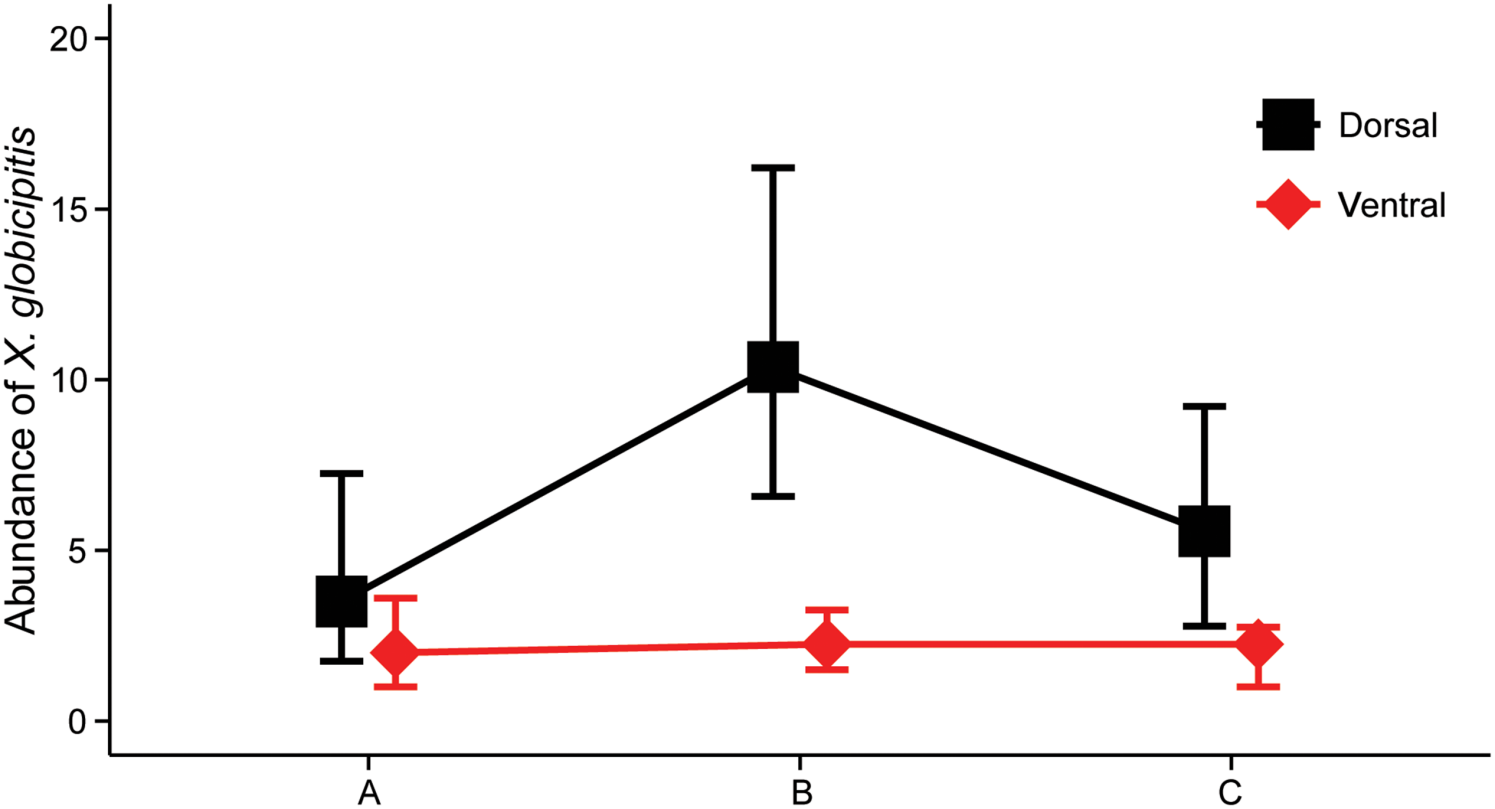
Average abundance of *Xenobalanus globicipitis* on caudal fin. Dorsal side (squares) and ventral side (diamonds) of three sections of the caudal fin of striped dolphins (see also Fig 3). Bars represent the 95% confidence interval.

In 58 dolphins positive for *X*. *globicipitis*, the frequency of occurrence was higher on the dorsal side than on the ventral side of the flukes (91.4% vs. 48.3%), with a mean abundance (95% CI) of 11.9 (8.2–18.5) and 2.5 (1.5–5.3), respectively; the difference was significant (Wilcoxon test, *Z* = 6.34, 1 d.f., p< 0.0001). Mean abundance per fluke section is shown in Fig 7. Both on the dorsal (Friedman test, χ^2^ = 14.21, 2 d.f., p<0.001) and ventral (χ^2^ = 10.88, 2 d.f., p = 0.004) sides, the abundance of *X*. *globicipitis* differed significantly among segments. On both sides, *post hoc* comparisons indicated that abundance on the central section significantly differed (p< 0.05) from those on lateral ones, which did not differ from one another.

Data on average median shell diameter for population segments of *X*. *globicipitis* on fluke sections is shown in Table 2. None of the main factors analyzed had significant effects on shell-size: ‘side’ (F_(1, 53)_ = 1.187, p = 0.281); ‘segment’ (F_(2, 53)_ = 0.386, p = 0.682); ‘dolphin type’ (F_(1, 53)_ = 0.070, p = 0.792); or ‘log_10_-abundance’ (F_(1, 53)_ = 2.373, p = 0.129).

**Table 2:** Average values of median shell diameter (S.D.) of *Xenobalanus globicipitis* on the dorsal and ventral sides of 3 sections of the flukes (see Fig 3) of the striped dolphin, *Stenella coeruleoalba*, from the western Mediterranean Sea.

Side	Section		
	A	B	C
Dorsal	2.11 (1.00)	2.66 (1.22)	2.43 (1.41)
	n = 8	n = 19	n = 9
Ventral	2.89 (1.25)	2.54 (1.18)	3.39 (0.79)
	n = 5	n = 12	n = 4

‘n’: sample size of dolphins.

Except for two cases, the posterior side of the shell was oriented to the quadrant 0° in all individuals of *X*. *globicipitis* (n = 63 from 21 dolphins) (see Figs 1 and 4). In other words, the cirral fan tended to be oriented opposite to the fluke edge. The two atypical specimens, both of which were allocated in densely populated segments, were oriented towards a lateral quadrant.

### Discussion

This study is based on a sample of dolphins that were found stranded dead. An important question is to what extent the patterns of habitat selection that we drew are valid for assessing the free-ranging dolphin population. In the study area, most stranding events of striped dolphins are associated to interactions with fisheries or disease [20]. Our particular sample contained a substantial number of dolphins that were killed by a morbillivirus, the effects of which are purported to significantly increase the probability of settlement of *X*. *globicipitis* [6, 20]. However, we found no significant difference in any of the comparisons between dolphins killed by the morbillivirus and dolphins stranded from other causes, suggesting that the basic pattern of habitat selection by *X*. *globicipitis* is conserved in dolphins experiencing diverse conditions prior to death. Still, dolphins coming ashore could be already dead at sea, providing additional opportunities for individuals of *X*. *globicipitis* to settle. It is unclear whether *X*. *globicipitis* can attach to cetacean carcasses, but we believe that this phenomenon would tend to blur patterns (e.g., erode patterns of differential settlement between fins), rather than create them. In addition, all the patterns found in this study can be related to meaningful ecological processes as we discuss later.

At the most inclusive scale, individuals of *X*. *globicipitis* were found exclusively on the fins, similarly as in previous studies on both stranded and free-ranging cetaceans ([15] and references therein). Rather interestingly, video records of wild dolphins show distribution patterns of *X*. *globicipitis* strikingly similar to those found in the present study (S1 Video). Apparently, this habitat-selection behavior is favored by the mechanical and trophic advantages associated to these sites ([9], see below). However, how individuals of *X*. *globicipitis* end up colonizing only these specific sites is an open, interesting question. In other coronulids, there is evidence that the cyprid larvae are able to chemically recognize cetacean skin as the correct substratum to settle and metamorphose ([7], see also [13] and [32]). We postulate a similar mechanism of host recognition in the case of *X*. *globicipitis*; however, exclusive settlement on fins is likely not chemically-mediated because the skin on fins does not seem to differ from that covering other body parts [33, 34]. A possibility is that larvae randomly contact any point on the dolphin’s body, then crawl to the trailing edge of fins using flow patterns as a physical cue. The ability of barnacle larvae to respond to local hydrodynamic conditions to select suitable settlement sites has been proven by both experimental and field data [35, 36]. However, given the small body size of the cyprid (approximately 1 mm for allied species of *X*. *globicipitis* [7]), this mechanism could work only for short distances, i.e., for larvae that selected settle points close to the trailing edge of fins (see below). On the other hand, there are reports of substantial post-settlement locomotion of epibiont barnacles of turtles that search for habitats with suitable flow [37]. However, the mode of attachment of *X*. *globicipitis* should preclude locomotion once the animal is settled [8, 9].

We hypothesize that the key factors that restrict the distribution of *X*. *globicipitis* to fins are an increased passive recruitment or decreased early cyprid mortality on these sites. Attachment success of barnacle larvae is determined by the velocity gradient over a solid surface, and cyprids fail to attach to areas of strong water flow [11, 38, 39]. Dolphins experience fast unidirectional flow over the body except on the fins, which function as hydrofoils that create transversal bound vortices starting from the leading edge, i.e., leading edge vortices [40–43]. Vortical flow over the fin surface produces two effects, i.e., (i) it increases the time that a body of water is in contact with the fin and (ii) moves water against the skin, thus promoting contact of larvae with the substrate. A higher contact rate is directly related with settlement rate for the barnacle cypris [44]. Perhaps larvae can also target other body parts when dolphins swim at low speed (e.g., when they sleep [45]), but they could likely be detached when the dolphin awakes and resume cruise speed. In summary, larvae of *X*. *globicipitis* should have a preferential contact with fins, greater chances of survival there, or both. On the other hand, water vortices are shed at the trailing edge [46], providing an ideal attachment site for a filtering organism living on a fast-swimming host [8]. We can postulate migration towards the trailing edge, perhaps being triggered by the vortical system. Also, once an individual barnacle attaches to the trailing edge, it could produce local eddies with reduced velocity gradients, enhacing settlement of other larvae nearby [38, 47].

At a finer scale, our results clearly show that the flukes were more frequently colonized by *X*. *globicipitis* than the other fins, and they harbored a significantly greater number of barnacles. This pattern does not appear to result from size differences in an available habitat for attachment. However, dolphins use each fin for different functions, i.e., use the flukes as a propeller, flippers for lift and breaking, and dorsal fin to avoid longitudinal spins [48]. During swimming, the flukes produce thrust by an oscillating dorso-ventral movement, following a longer path per unit time and sweeping more volume of water than the other fins [49, 50]. These effects alone should increase the chances of colonization by larvae of *X*. *globicipitis*. Moreover, vortex formation is also more evident on the flukes. Apart from the transversal vortices associated with the hydrofoil function, which are common to all fins, the flukes use wake capture as a mechanism for enhanced production of lift and thrust. During a complete cycle of upstroke and downstroke, two vortices of opposite rotational sense are produced, and the flukes intercept them to generate lift [40, 51]. This means that the same body of water that has rolled over the fluke surface is further contacted during the oscillation (see [51] for details), thus increasing opportunities for larval contact. Interestingly, barnacles, after removing their potential density-dependent effects, were also significantly larger on the flukes than on the other fins. This suggests that water flow patterns generated by the caudal fin could be suitable for filtering, thus enhancing growth. In this context, individuals of *X*. *globicitipis* were attached so that the cirral fan was oriented opposite to the trailing edge, an orientation that is precisely that necessary to maximize contact with incoming water from the vortices [40, 51]. An active positioning of the barnacles against the main direction of flow has been observed in other barnacles [38, 52].

The distribution of *X*. *globicipitis* on the flukes was not random; a significantly higher frequency of settlement and abundance of barnacles was found on the dorsal side. There is no anatomical difference between fluke sides [33] that could account for this pattern. The most parsimonious explanation is that there is some sort of an asymmetrical performance of fluke oscillation. This, however, is a controversial isssue. In the bottlenosed dolphin, Parry [53] reported differences between stroke duration, and Videler and Kamermans [54] suggested that the downstroke represented the main propulsive force, while the upstroke acted as a secondary propeller used as a recovery mechanism. Apparently, oscillation also becomes more asymmetric, emphasizing the downstroke as speed increases [51]. In contrast, Fish and Rohr [40] argued that caudal oscillation was symmetrical, allowing production of equal thrust by both up- and downstrokes. The distribution pattern of *X*. *globicipitis* is compatible with the hypothesis of an asymmetrical oscillation. Studies by Ashraf et al. [55] on the hydrodynamics of flapping foils detected leading edge vortices (LEV) on the opposite side of the movement. If downstroke is enhanced, greater LEVs would be created on the dorsal side, increasing contact rate of larvae [56] and, perhaps, offering more suitable conditions for filtering at the trailing edge. Additionally, the barnacle’s body bends at each fluke stroke, and therefore individuals settled on the ventral side would suffer greater mechanical stress. If the cyprids of *X*. *globicipitis* exhibited crawling ability, they could move from the ventral to the dorsal side at the trailing edge.

Finally, *X*. *globicipitis* preferentially occupied the middle area of the trailing edge of the flukes. Little is known about the local water flow dynamics on the fluke, but such findings about local characteristics could help advance a preliminary hypothesis to explain this pattern. The collagen-based caudal fin is not rigid; structural flexibility creates passive cambering in the oposite direction of the stroke, moving the edge and tips of the flukes upward during the downstroke and downward during the upstroke [57, 58]. This passive bending is created both spanwise and chordwise and helps to prevent the loss of thrust during the end of each stroke [57]. The camber is specially predominant toward the middle of the edge, while the tips are less affected by the flow forces [59]. In the central section, the notch divides the caudal fin into two equal parts, creating an 'interruption' in the trailing edge line. We speculate that this break might modify the local hydrodynamics, allowing water from the camber to preferentially flow through the notch, both increasing in this area the likelihood of contact by larvae of *X*. *globicipitis* and increasing the filtering performance of adults.

In summary, we postulate that habitat selection by *X*. *globicipitis* on small cetaceans results from the following proceses: (1) chemical recognition of the cetacean as an acceptable substratum on which to settle; (2) passive selection of fins due to the creation of a vortex at these sites that increases contact with skin and provides early survival of larvae; (3) potential migration of larvae to the trailing edge, ultimately increasing the filtration performance as adults; (4) passive selection of the caudal fin due to a specific vortical flow system that enhances more contact by larvae when compared with that on other fins; (5) active orientation against the main direction of flow on the flukes, i.e., facing the trailing edge; (6) attachment on the dorsal side of the flukes, possibly associated to asymmetrical oscillation of these appendages, and (7) maximal occurrence on the central segment of the flukes, perhaps because of the most suitable water flow conditions generated by the fluke performance for both settlement and filtration.

## Supporting Information

S1 Video Movie of wild short-beaked common dolphins, *Delphinus delphis*, with *Xenobalanus globicipitis*. Video showing distribution patterns of *X*. *globicipitis* similar to the ones described in the present paper. Reprinted from https://www.youtube.com/watch?v=8aJdW5IRZSs under a CC BY license, with permission from Tim Hammond, original copyright 2013.
(MP4)
